# Evaluation of Water Status and Thermal Characteristics of Dried Carrot Half-Slices in Correlation with Physicochemical and Sensory Properties

**DOI:** 10.3390/molecules31111789

**Published:** 2026-05-22

**Authors:** Anna Ignaczak, Łukasz Woźniak, Mariola Kozłowska, Hanna Kowalska

**Affiliations:** 1 Department of Food Engineering, Institute of Food Sciences, Warsaw University of Life Sciences, 159c Nowoursynowska St., 02-776 Warsaw, Poland; 2 Department of Food Safety and Chemical Analysis, Institute of Agricultural and Food Biotechnology—State Research Institute, 36 Rakowiecka St., 02-532 Warsaw, Poland; lukasz.wozniak@ibprs.pl; 3 Department of Chemistry, Institute of Food Sciences, Warsaw University of Life Sciences, 159c Nowoursynowska St., 02-776 Warsaw, Poland; mariola_kozlowska@sggw.edu.pl

**Keywords:** vegetable snacks, enriching, microwave-vacuum drying, water stability, thermal properties, FTIR, microstructure, sensory quality, polyphenols, antioxidant activity

## Abstract

The aim of the study was to investigate the effect of enriching carrot slices by NFC (not from concentrate) juices from chokeberry (CH), sea buckthorn (SB), cherry (CHE) and carrot (CA) before microwave-vacuum (MVD) and freeze-drying (FD) carrot on the physicochemical and thermal properties. While water activity (AW) was not dependent on enrichment treatment but only on drying method, NFC juices significantly enriched carrot slices with biocomponents. Freeze-dried samples, as a reference, had significantly lower AW than those dried by the MVD method. Both FD and MVD-dried samples had comparable polyphenol content and DPPH antioxidant activity (AA), but the MVD-dried samples exhibited higher ABTS antioxidant activity. Carrot enrichment in chokeberry and cherry juices resulted in up to six and 10 times higher TPC than in the raw material. In addition, samples enriched in these juices and dried with FD proved to be the most stable in terms of water state and glass transition temperature (61.4 and 69.6 °C) and water activity (approx. 0.10). In FTIR analysis, all samples exhibited similar spectral shapes, indicating similar chemical composition and functional group composition. Only in the spectral region below 900 cm^−1^ were unique molecular vibrations induced by various organic compounds present. Enriching carrot in juices and MVD can lead to increased hardness (Fmax and breaking work), although this is associated with increased crispness, resulting from the microstructure with a large number of small pores, especially in MVD samples enriched with cherry, chokeberry, and carrot juices, with scores of 8.0–8.4 In this respect, the average crispness rating of the MVD samples (7.2) exceeded that of the FD samples (6.8). If there is a requirement for crunchiness in the future production of dried vegetables as snacks, changes in hardness should be prioritized, along with color and biocomponent content.

## 1. Introduction

According to Global Market Insights Inc. [1], the healthy snack market is growing rapidly. It is estimated that the market will reach USD 185.6 billion by 2034, compared to approximately USD 101.3 billion in 2024. Dried vegetable snacks with high nutritional value and sensory appeal, particularly in terms of crunchiness, are gaining popularity among consumers. A significant feature of such snacks is their easy availability. Carrots are a well-known vegetable consumed in various forms, both fresh and processed, including dried vegetable snacks, which occupy a niche market and are valued for their nutritional value, including a high content of carotenoids with relatively high thermal stability. The vegetable is a rich source of many vitamins, vitamin A and its precursor (β-carotene), vitamins C and E, and the B vitamins, as well as minerals such as calcium, iron, potassium, magnesium, phosphorus, and sodium [2,3]. Consuming carrots can benefit eye health, lower cholesterol levels, and improve digestion [4]. Therefore, carrots are a useful raw material for the production of dried snacks, also due to the simplicity of the technological process, and the need to increase vegetable consumption may increase the interest of researchers and consumers.

Among various techniques, microwave and reduced-pressure drying have been investigated for producing dried carrot products, with varying degrees of success [5,6,7,8]. Before microwave-vacuum drying, preliminary moisture reduction is typically achieved by convection; similarly, this was accomplished before instant controlled pressure drop drying of peaches [9]. Figiel and Michalska [10] demonstrated that a convection drying step enables partial water evaporation at a lower temperature, reducing sample weight and tissue damage during the subsequent microwave-vacuum drying step. Furthermore, because microwave drying provides volumetric heating, field unevenness often leads to local overheating or underheating, which ultimately affects product quality [11,12]. However, it has been demonstrated that despite losses or degradation during processing, dried plant products remain a valuable source of bioactive compounds and antioxidants [13]. Wardhani et al. [14] found that microwave-vacuum drying can reduce undesirable sensory changes and nutrient losses that can occur with longer drying times or high surface temperatures. As a result, dried fruits were similar to those dried by freeze-drying, with slight color changes and limited losses of bioactive components. Their retention is a compromise between economically viable drying conditions and gentle processing techniques to preserve both nutritional value and sensory quality [15]. In this regard, numerous studies have investigated the impregnation of plant materials with various ingredients, typically in isotonic solutions and under reduced pressure conditions [16,17,18] or through pre-osmotic dehydration [19]. Osmotic pre-dehydration was shown to have a beneficial effect on the textural properties of fruit chips. Various osmotic substances, particularly sugars, were found to penetrate the tissue of the dehydrated material, facilitating volume expansion, the formation of a uniform microstructure, and thermal properties [9,20]. Few studies have examined osmotic enrichment (OE) in solutions with concentrations slightly higher than the concentration of tissue cell sap. It has been proven that osmotic enrichment of sample carrots in NFC (not from concentrate) juice ingredients before drying has been proven to be highly beneficial in effectively increasing the content of desirable juice components, thereby preserving nutritional value and health-promoting properties, while also imparting attractive sensory characteristics without the use of high temperatures [5,6]. However, the properties of such dried materials have not been studied with respect to their microstructural and thermal properties.

The pre-treatment and drying processes of carrots, as well as their nutritional and sensory quality, depend on the water status of this type of food. Water is one of the most important ingredients of food, as it significantly affects the shape, quality, sensory properties, as well as stability and safety, including shelf life. Using water vapor adsorption isotherms for dried foods allows for determining appropriate processing and storage conditions. Sorption isotherms are widely used and described mathematically [21,22,23]. However, using different models is associated with various limitations. It is challenging to develop a theoretically sound and universally applicable model for the full range of water activity. Determining the water status of food also includes moisture content, water activity (AW), and the mobility of water molecules. The stability and quality of dried products depend on these indicators in various ways. Moisture content alone is insufficient to predict a product’s stability; it is necessary to understand its water activity. Water activity exhibits thermodynamic properties resulting from the interactions between water molecules and the food matrix [24]. Water activity reflects its availability, and exceeding a value of 0.6—the conventional threshold for dried products—allows for the growth of microorganisms and chemical reactions that can affect food quality. Therefore, this indicator is included as an important food safety indicator in many government regulations, including the Codex Alimentarius and European Union Regulation 2073/2005 [25]. Low moisture levels, typically up to 15%, and low water activity are adequate to maintain the crisp structure of dried foods. However, a product may be unstable at low moisture but relatively high-water activity. Even products with similar moisture content can vary significantly in stability and spoilage susceptibility [25]. Furthermore, the mobility of water molecules is influenced by glass transition phenomena and the tendency to plasticize, both of which play a key role in determining textural properties and quality deterioration. Plant tissue, as a complex food matrix, undergoes various changes during technological processes, making it difficult to draw simple conclusions about its stability. Therefore, understanding the effect of various process conditions, including pre-enrichment, on the water status of dried products with desired properties is crucial. Dried products typically occur in a glassy, amorphous form that maintains its stability during storage [26]. Assessing the stability of amorphous materials between the glassy and highly elastic states, the glass transition temperature (Tg) is considered, which can change due to molecular motion at the microscopic level resulting from processing technology and, consequently, changes in tissue behavior [9,27]. There are relatively few comprehensive reports on microwave drying to produce high-quality, health-promoting, and sensory-friendly vegetable snacks, along with explanations for changes in Tg of dried products, including carrots, in relation to water status, microstructure, and texture-forming mechanisms.

Regarding dried vegetable snacks, previous studies have focused on single aspects related to drying efficiency, nutrient retention, or selected sensory attributes. In science, there is a research gap in comprehensive analyses that combine issues related to water status, thermal properties, microstructural changes, and sensory properties of dried carrot snacks. Therefore, the drying method and the potential of NFC juices to enrich carrot slices will be the subject of research aimed at explaining the above issues, and in particular at demonstrating the effect of enrichment on the content of bioactive components, such as polyphenolic compounds and antioxidant properties, as well as the possibility of shaping the sensory quality and stability of dried products. Meeting current trends, this research may contribute to the wider adoption of vegetable drying methods with pre-enrichment using NFC juices, which may be of interest to potential producers of snacks with desirable properties and increased bioactive ingredient content.

## 2. Results

**Figure 1:**
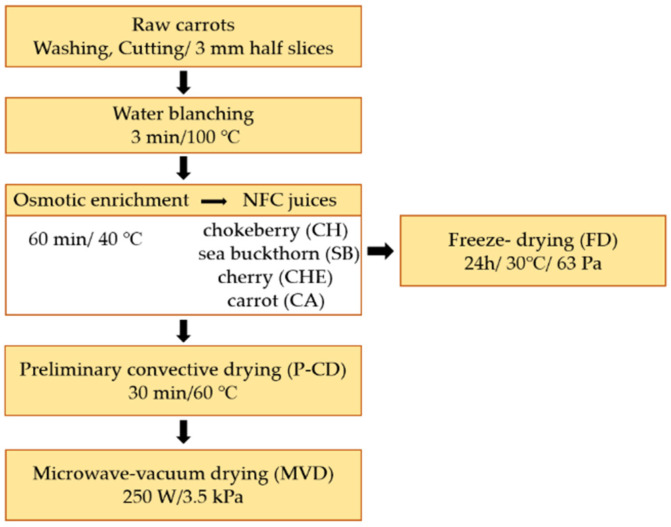
Scheme of the experimental procedure for drying carrots.

To assess the water status in dried carrot, a research scope was planned, including the production of samples in the shape of half-slices with an initial thickness of 3 mm, which were blanched in boiling water, osmotic enriched in NFC (not from concentrate) juices, and then dried using the hybrid method by convection (according to the manufacturer’s instructions) and final microwave-vacuum drying (Figure 1). Dried carrots were assessed for their physicochemical properties and sensory characteristics, and correlation and principal component analysis (PCA) were performed. In the following research stage, the effect of low moisture on microstructure changes was assessed, and the thermodynamic properties of dried carrot samples were examined.

### 2.1. Physicochemical Properties of Dried Carrot

#### 2.1.1. Dry Matter Content (DM), Water Activity (AW), Polyphenol Content and Antioxidant Activity (DPPH and ABTS)

Carrot samples dried by the MVD method showed significantly lower dry matter content (DM) values, but higher water activity (AW) and ABTS antioxidant activity than those obtained by freeze-drying (Table 1).

There was no effect of the drying method on the total polyphenol content (TPC) or antioxidant activity (AA) of carrots determined by the DPPH method. At the same time, the mean DM values of the MVD-dried samples were approximately 91.4%, and of the freeze-dried ones 93.7%. However, the mean water activity of the freeze-dried samples was approximately 3.5 times lower, ranging from 0.092 to 0.159. Enrichment of carrots with chokeberry juice resulted in statistically the highest DM (95.6–96.9%) but did not affect the water activity of the dried carrots. Although all samples were sufficiently dried and ensured microbiological stability [28], freeze-drying was more advantageous due to its significantly lower water activity; however, it was less favorable due to the antioxidant activity of ABTS. The obtained results indicate that the effects of microwave-vacuum drying of carrots, in terms of DM and AW, are lower compared to those of freeze-dried materials, but comparable in terms of polyphenol content and antioxidant activity. According to Zeng et al. [29], the accelerated heating process of the material using the MVD method promotes the inactivation of oxidative enzymes, which may increase the preservation of phenolic compounds. Similarly, freeze-drying of osmotically dehydrated strawberries showed only slightly higher polyphenol content and DPPH• radical scavenging capacity compared to those dried using the microwave-convection method (MCD) [30]. These differences may result from the lower drying temperature of FD. However, high temperature, related to the power of MCD or MVD microwaves, may cause thermal degradation and oxidation of biocomponents [31] due to uneven heating with local overheating, even with a much shorter process duration.

The total polyphenol content in fresh carrots was approximately 236 mg/GAE 100 g d.m. Similarly, in the study by Amin et al. [31], fresh carrots (*Daucus carota* L.) were approximately 189 mg GAE/100 g. However, compared with the study by Amin et al. [31], in which microwave drying at 170–510 W decreased TPC, our study showed an increase in TPC. This could have been due to the lack of treatment with a 0.25% potassium metabisulfite solution for 20 min, which could have led to leaching of these components. It should be emphasized that the TPC was approximately 5-fold higher as a result of the relatively short MVD method compared to freeze-drying (FD), which is considered to be the method that best preserves biocomponents [32], resulting in only a 3-fold increase in TPC compared to the content in fresh carrots (Table 1). The FD method also resulted in a greater reduction in antioxidant activity (DPPH). This may be due to the limited heat transfer in the thermal medium under FD conditions, resulting in a significantly longer drying time [33]. The considerably shorter MVD method exposed carrot samples to short-term elevated-temperature exposure under reduced pressure. A significant effect of carrot enrichment with NFC juices on the increase in total polyphenol content (TPC) was demonstrated, with the greatest effect observed in the case of samples treated with chokeberry juice, which resulted in approximately 10 times higher polyphenol content than in the raw material, and cherry juice, which resulted in approximately six times higher polyphenol content than in the fresh carrot. Simultaneously, enrichment expressed TPC of dried carrots correlated highly positively with DPPH antioxidant activity (*R*^2^ = 0.95), but lowly with ABTS (*R*^2^ = 0.35). Compared to the raw material, the DPPH antioxidant activity of dried carrots was varied (Table 1), particularly lower when fortification was applied in carrot juice. This can be explained by the fact that, unlike pasteurized NFC commercial juices, freshly pressed carrot juice was not subjected to heat treatment; therefore, the enzymes present in the juice could affect the decomposition of components with antioxidant properties. Enrichment of chokeberry juice resulted in the highest DPPH values of 6.4–7.5 mg Trolox/g d.m., which were 23–44% higher than in the raw material. However, the ABTS values of samples enriched with chokeberry and cherry juices were comparable to those in the raw material (4.5–4.6 mg Trolox/g d.m.). As reported by Boudebbaz et al. [34], the DPPH and ABTS tests differ in their mechanisms; DPPH involves electron transfer, while the ABTS test encompasses both electron and proton transfer, and is therefore considered more versatile. This is also due to the solubility of the ABTS reagent in aqueous and organic solvents, making this test useful for assessing the antioxidant properties of substances in both hydrophilic and lipophilic environments and over a wide pH range. Furthermore, DPPD reacts best with small molecules, while ABTS reacts with a wider range of structures, including large and hydrophilic molecules. However, the lack of significantly higher ABTS values than DPPH in the obtained results (Table 1) may be due to the presence of various substances exhibiting antioxidant activity and other factors, such as pH, solubility, and structural form, which may hinder the extraction of these substances from the tested samples. These results could be confounded by other mechanisms. For example, Li et al. [35] found that during oxidation, spontaneously generated radicals can be stabilized by intramolecular hydrogen bonds or semiquinone radicals, disrupting the free radical chain reaction.

#### 2.1.2. Sorption Properties

Due to the diverse properties of dried material related to the adsorption or desorption of water vapor from the environment, it is recommended to examine the hygroscopic properties (HG) of the dried material, as they affect the product quality [36], especially during storage [37]. Each product has a so-called critical water content, exceeding which, due to water vapor sorption, unfavorable changes occur in its physical, physicochemical, microbiological, and enzymatic quality characteristics [38]. The sorption mechanism depends on the physical properties of the material, especially porosity, as well as its chemical composition [39].

**Figure 2:**
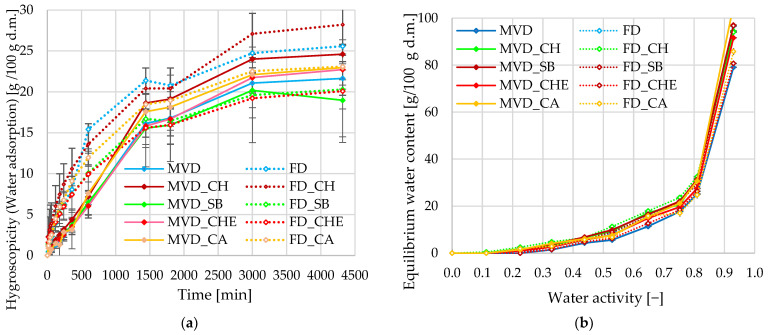
Effect of drying method and enrichment on: (**a**) hygroscopicity (HG) and (**b**) sorption isotherms of dried carrot (WCeq) at 25 °C.

The effect of enriching dried carrots using the MVD method, compared to the FD method, on the moisture adsorption of samples tested at AW = 0.753 is presented in Figure 2a. Due to large standard deviations, no significant differences in the hygroscopicity of the dried material were observed, regardless of the enrichment treatment used (Table 2). However, the effects of the drying method and test duration, as well as their interaction with time, were significant over the 0–4320 min (3 days) range. Assessing the trends in terms of the effect of pre-enrichment of carrot slices, calculations of the mean values from the HG curves allowed this study to conclude that untreated MVD dried carrot slices were at the beginning of the lower water vapor adsorption values (approx. 7.16 g/100 g d.m.), while untreated FD were at the end of this range (approx. 10.64 g/100 g d.m.). The enriched dried carrot had an increasing order of adsorption from SB (approx. 7.83 g/100 g d.m.), CHE (approx. 8.01 g/100 g d.m.), CA (approx. 8.80 g/100 g d.m.), to CH (approx. 10.25 g/100 g d.m.). Analyzing the effect of the drying method, higher hygroscopicity was observed for freeze-dried (FD) samples, with an average of 17.81 g/100 g d.m. across the entire drying process, compared to 16.74 g/100 g d.m. for MVD samples. These differences were most noticeable within the first 24 h. This is due to the characteristic high porosity of samples produced by the freeze-drying process. Relatively high HG was observed in MVD_CH and FD_CH samples enriched with chokeberry juice, with values similar to, and at the final stage even exceeding, those of the FD control samples. This could be due to the properties of the juice components that penetrated the carrots, including FD_CH. Regardless of the drying method, enriching carrots with sea buckthorn juice resulted in the lowest values of this indicator. The hygroscopicity curves of dried carrot slices enriched with carrot juice, especially after longer times (>24 h), showed intermediate HG values.

Moisture sorption isotherms, i.e., changes in equilibrium water content (WCeq) as a function of water activity (AW), reflect water binding by the dried material and enable the determination of its stability depending on ambient humidity during storage [40]. Analyzing the overall course of sorption isotherms, a significant effect of NFC enrichment in juices and water activity, as well as their interaction, was demonstrated, while no effect of the drying method was observed (Figure 2b, Table 2).

Similar to the hygroscopicity of carrot samples, the lower average equilibrium water content calculated for the entire isotherm was observed in the MVD control samples (14.48 g/100 g d.m.), while the highest was found in samples enriched with sea buckthorn (18.61 g/100 g d.m.). A significant effect of drying method was also found, but the effect of pretreatment was greater. This is confirmed by the fact that samples enriched with carrot juice (CA), chokeberry (CH), and freeze-dried without enrichment (FD; 18.11 g/100 g d.m.) showed quite similar values in the range of 17.65–18.96 g/100 g d.m. (Table 2, Figure 2b). Statistical analysis of repeated-measures designs used to examine differences in the course of individual isotherms of dried carrot slices showed significant differences in equilibrium moisture content at each water activity level (Table 2), yielding 10 homogeneous groups.

However, the most pronounced differences occurred in the AW range from 0.50 to 0.75 (Figure 2b). To achieve stable dried carrots using the MVD method, with or without enrichment, the drying process should reduce the moisture content to below 10%. Compared to our study (Figure 2b), the study by Eim et al. [22] showed similar equilibrium water contents in convectively dried carrot slices tested in the range of 10–50 °C and similarly in carrots dried by other methods [41] and Iaccheri et al. [23]. At low activities, water sorption is mainly due to the presence of biopolymers; at higher AW, water is absorbed by low molecular weight components [21]. In the 0.65–0.80 range, there was a significant increase in hygroscopicity, reflecting the transition from single-layer to multi-layer water binding and changes in water-binding mechanisms caused by enrichment in NFC juices. Increasing water content loosens the structure and exposes new adsorption sites, thus increasing the adsorption capacity. Sorption isotherms of enriched samples did not correlate with hygroscopicity, likely due to changes in the dried product components during long-term storage. Dried products, in which water removal is rapid, and the increase in viscosity prevents crystallization, are in an amorphous state at room temperature [42]. Water induces a transition to a viscoelastic state, in which molecular mobility is significantly greater, leading to chemical changes, including accelerated crystallization of the components [43,44].

According to the BET classification, the observed isotherm shape of dried carrots was type III (Figure 2b), consistent with the shape observed for dried carrots in the study by Iaccheri et al. [23]. According to the theory [22,23], this type of isotherm is typically associated with a matrix containing small molecules, such as sugars and salts, that are available for interaction with moisture. In the literature, this is explained by changes in the structure of these molecules and an increase in the number of active water-binding sites, also as a result of swelling of hydrophilic biopolymers, such as proteins and polysaccharides [21,22]. Experimental data were analyzed using BET, GAB, Lewicki, Oswin, and Henderson models (Table 3), which were evaluated based on the coefficient of determination (*R*^2^), the percent root mean square error (*RMSE*), and the residual coefficient of variation (*Ve*). A good model fit was observed in terms of the coefficient of determination, with *R*^2^ > 0.99 in most models, and Ve, with values in the range of 0–20% considered acceptable [41]. According to theoretical assumptions, the BET model is suitable for low water activities. However, it proved to be as useful as most other models due to high *R*^2^ values, most of which were above 0.995; only the Henderson model had values in the range 0.987–0.998. The good model fit is also evidenced by low Ve values (5.3–14.6) and *RMSE* values (0.67–2.48). BET and GAB models have been frequently used to describe food sorption isotherms. Despite the theoretical limit of approximately 0.4 water activity, BET adsorption analysis is particularly useful because it relies on the monolayer concept, which represents the optimal moisture content a product should achieve and maintain to minimize adverse reactions during storage. The monolayer capacity, determined by the BET equation, ranged from 6.059 to 8.124 g/100 g d.m. Higher values, indicating higher stability, were observed in samples dried by freeze-drying. Among the enriched samples, higher values were found in samples treated with carrot juice (MVD_CA), especially after MVD (approx. 8.12 g/100 g d.m.), and slightly lower (6.98–7.28 approx.) in samples enriched with chokeberry and sea buckthorn juice dried by both methods (MVD_CH, FD_CH, MVD_SB, FD_SB), however, comparable to the freeze-dried control sample (approx. 7.28).

#### 2.1.3. Structure and Microstructure Analysis

Table 4 presents illustrative photos of dried carrot samples taken at the same distance, images of the internal microstructure obtained with an electron microscope at 500× magnification, and reconstructed images of the three-dimensional microstructure obtained with a µCT microtomography of microwave-vacuum and freeze-dried carrots, depending on osmotic enrichment in NFC juices. In general, drying thin carrot slices resulted in changes in tissue structure, which appeared quite compact in SEM images, but high porosity was observed in µCT analysis (Table 4 and Table 5). In the case of microwave drying, volumetric heating leads to rapid vapor formation within the tissue, generating an internal pressure gradient that ruptures cell walls and forms diffusion channels [45]. In the study by Smith et al. [46], the original carrot microstructure (SEM) was lost after vacuum drying, resulting in collapsed, compressed cells, but structures were still recognizable on the outer surface. Such changes result from faster water removal from the outer surface than from the inner surface, leading to greater cell damage and densification. According to Mondal et al. [47], freeze-dried products retain the uniform structure of carrots. Probably due to the thin slice thickness, moisture loss caused visible collapse of the cell walls, and in some cases, the occurrence of quite large intercellular spaces in the shape of cavities was also observed. Wang et al. [9] showed that osmotic pre-dehydration of peach chips inhibited the formation of large pit structures in microstructural images.

Considering the great difficulty in interpreting the presented SEM images, the use of advanced software for microstructure interpretation of µCT microtomography images becomes extremely useful [48]. µCT microstructure analysis is particularly important for obtaining information on the microstructure parameters of dried products [49]. The possibility of observing the same fragment size across all samples in a 3D system and of determining a large number of microstructure indices is highly beneficial.

The drying method had a significantly greater effect on carrot microstructure than the enrichment treatment. The lack of significance of enrichment on some parameters is due to the dominant effect of drying. It may also be due to the high parameter standard deviation, resulting from significant microstructural variation. Compared to freeze-drying, microwave-vacuum drying resulted in significantly lower values for percent object volume, structure thickness, and structure model thickness, but significantly higher values for total porosity and object surface to volume ratio (Table 5). All types of dried carrot slices exhibited a porous microstructure. The higher POV percentage for the freeze-dried samples indicates a greater proportion of solid substances in the sample volume. Significantly higher values of the structure model index indicate differentiated pore shapes compared to MVD-dried samples. However, positive SMI values for all samples indicate surface convexity and the absence of concavity, as an ideal plate, cylinder, and sphere have SMI values of 0, three, and four [50]. The SMI values of the carrot samples were related to the plate (0.58–1.15 SMI), while pores closest to the cylinder shape (rod-shaped structures) were exhibited by freeze-dried samples without enrichment (2.25) and enriched in carrot juice (1.63). Porosity, which is a measure of the ratio of pore volume to the total volume of the material, is one of the parameters affecting the texture, mechanics, and quality of dried food products [49]. The total porosity of the freeze-dried samples ranged from 66.3 to 85.7%, and of the MVD samples from 71.0 to 92.6%. Regardless of the drying method, the samples enriched with chokeberry had the lowest porosity (66.3–71.0%). Among the freeze-dried samples, the highest porosity was observed in the sea buckthorn juice-enriched samples (85.7%) and in the carrot juice-enriched MVD samples (92.6%). Closed porosity was very low, ranging from 0.01 to 0.17% and statistically insignificant. Considering that open porosity is the difference between total and closed porosity, the dried carrot samples were characterized by open porosity. High values, with a quite uniform ranking from 82.4 to 134.4 mm^−1^, for the object surface to volume ratio (OSVR) indicate a large number of small pores in the dried carrot slices (Table 5). In the study by Siebert et al. [51], carrot disks dried by the FD method retained small pores, similar to those in fresh carrot tissue, while those dried by the MVD method retained large pores. They explained the occurrence of these large pores as microstructure swelling induced by the microwave electromagnetic field during volume drying.

The lowest values were found in samples freeze-dried without enrichment, and enrichment resulted in greater variation in OSVR values compared to MVD-dried samples. It should be emphasized that, regardless of NFC juice, pre-enrichment of carrot slices and MVD resulted in increased OSVR, leading to a more uniform structure with a large number of small pores and increased total porosity, thereby reducing the number of large pores (cavities). As demonstrated by Prawiranto et al. [27], the selection and optimization of drying conditions can be explained and optimized based on changes in the microstructure of the dried products. In the case of apple tissue dried using the convective and irradiation-convective methods, the former demonstrated the formation of a layer of increased porosity relative to the initial porosity, and the latter showed a layer of deformed cells near the sample surface, associated with shrinkage and deformation of individual cells. This may explain the diversity of the structure of the dried products.

#### 2.1.4. Glass Transition Temperature (Tg)

**Figure 3:**
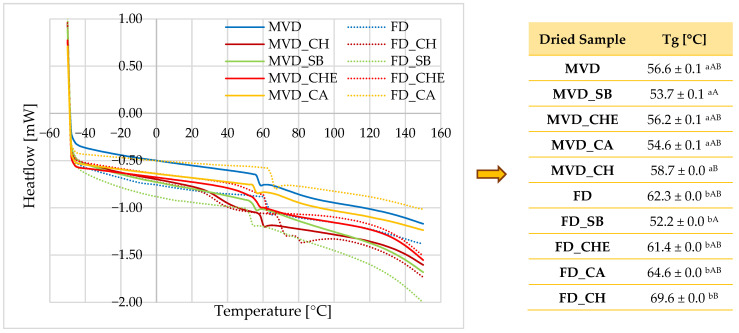
Effect of drying method and enrichment in NFC juice on glass transition temperature (Tg). Explanations: homogeneous groups at *p* = 0.05: ^a^, ^b^—effect of drying method: MVD—microwave-vacuum drying; FD—freeze-drying; ^A^, ^B^—effect of osmotic enrichment in NFC juices: CH—chokeberry, SB—sea buckthorn, CHE—cherry, CA—carrot.

Differential scanning calorimetry (DSC) was used to determine the glass transition temperature (Tg), below which materials should be stored. Temperatures in the obtained dried materials ranged from 53.7 to 58.7 °C for MVD dried materials and from 52.2 to 69.6 °C for FD dried materials, whose mean Tg value (56.0 and 62.0 °C, respectively) was significantly higher for FD, and therefore more favorable for sample stability and durability (Figure 3). Enrichment of carrots with chokeberry juice contributed to the statistically highest Tg among both freeze-dried (69.6 °C) and microwave-vacuum-dried (58.7 °C) samples, while sea buckthorn had the lowest, 52.2–53.7 °C. However, the hygroscopicity of both enriched dried materials was similar and relatively the lowest (Figure 2a). As stated by Wang et al. [52], changes in water state in plant tissues have a close correlation with the glass transition temperature (Tg), and the presence of low-molecular-weight sugars after osmotic pretreatment of white radish resulted in a decrease in the glass transition temperature (Tg) due to the lower crystallization temperature. Dry products obtained in most drying processes are usually in a glassy, amorphous form. For the product to be stable during storage, this physical state should not change. When the temperature is above the glass transition temperature (Tg), the amorphous solid exists in a “rubbery” viscoelastic state. In this state, the molecular mobility of the matrix and reactants is accelerated, leading to increased rates of physicochemical changes in the dried products, such as adhesion, caking, agglomeration, crystallization, loss of volatile substances, browning, and oxidation [53]. These changes play an important role in both the processing and storage of dried food products.

#### 2.1.5. Fourier-Transform Infrared Spectroscopy (FTIR)

**Figure 4:**
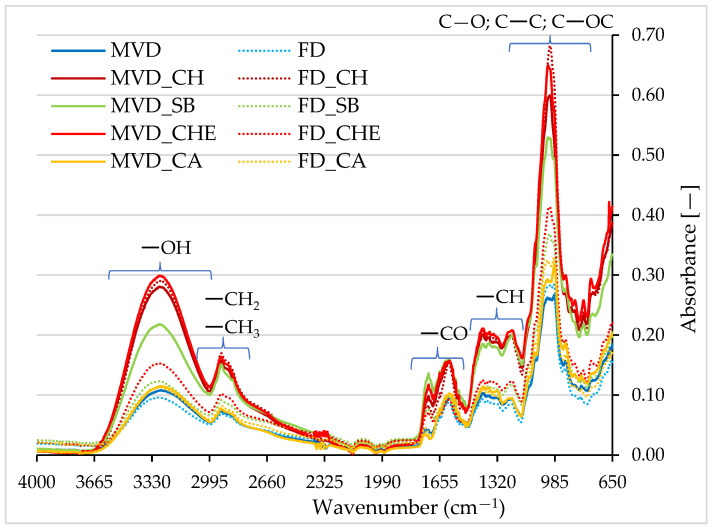
Fourier transform infrared (FTIR) spectra of microwave-vacuum and freeze-dried carrot, depending on drying method and osmotic enrichment in NFC juices. Code designations: MVD—microwave-vacuum drying; FD—freeze-drying; NFC juices: CH—chokeberry, SB—sea buckthorn, CHE—cherry, CA—carrot.

Fourier transform infrared spectroscopy (FTIR) is an analytical method that allows assessment of the chemical composition, including the identification of functional groups and intramolecular bonds, of interacting components of the analyzed material [54]. Infrared (IR) spectra obtained by FTIR analysis for dried carrot half-slices subjected to enrichment treatment in NFC juices are presented in Figure 4. The analysis of these samples was carried out in the wavenumber range of 650 to 4000 cm^−1^.

All tested samples exhibited a similar spectral shape, indicating a comparable distribution of functional groups. However, the peak intensities of the studied samples within the recorded infrared spectral wavenumber ranges varied, reflecting differences in the relative abundance of specific functional groups. The spectral wavenumber range of 3600–3000 cm^−1^ is associated with the stretching vibrations of the –OH, –NH, and –CH groups. The –OH bond vibrations in this region are primarily associated with the presence of water molecules in the tested material. However, they can also originate from the presence of phenols, carboxylic acids, or alcohols. The band visible in the absorbance range of 2950–2750 cm^−1^ corresponds to the presence of vibrations of the alkyl groups –CH_2_ and –CH_3_. The highest intensity in this region was observed for the FD_CHE sample. Vibrations in the wavelength range of 1800–1490 cm^−1^ are characteristic of the carbonyl group –CO bonds bound to the amide functional group. The spectral regions visible in the range 1480–1170 cm ^−1^ reflect the presence of –CH bonds present in cellulose and hemicellulose, the main polysaccharides that build the plant cell wall. The most intense spectrum was observed in the vibration region 1200–900 cm^−1^, which corresponds to C–O, C–C and C–O–C bonds. The spectral region below 900 cm^−1^ corresponds to the fingerprint region, providing information on complex molecular vibrations characteristic of specific organic compounds [55,56,57,58].

#### 2.1.6. Color Parameters and Sensory Evaluation

Color is a key factor in determining freshness, ripeness, and market appeal—traits that heavily influence consumer choices [31]. The color parameters of dried carrots depended on the drying method and osmotic enrichment in NFC juices (Table 6). Regarding the drying method, significant differences were mainly observed in lightness (L*) and redness. Dueik et al. [8] explained that the degradation of trans α- and trans β-carotene is directly related to the a* and b* parameters, which represent yellow and orange pigments, respectively. Samples dried using the FD method showed a notable increase in lightness and redness compared to the color of the raw material. These changes led to larger differences in the absolute value of the color difference ΔE compared to samples dried using the MVD method. Lightening of the color of freeze-dried carrots is typical and has been shown in many studies [5,59,60,61]. This is partly due to the change in structure related to high porosity, which affects the angle of light reflection [61], as well as the influence of lower temperature [61] and the absence of oxygen during drying, which effectively prevents browning caused by oxidation [52,62,63].

Enriching carrots before drying caused significantly greater changes in all color parameters, which were related to the content of color components in the individual juices and their penetration into the carrot samples. Using chokeberry juice resulted in the greatest reduction in color brightness (darkening), as well as in the a* and b* parameters and saturation C, especially in MVD-dried carrots. However, it increased the hue angle h, particularly in FD-dried carrots. The smallest color changes were observed when using carrot juice. Therefore, using carrot juice can be considered beneficial for maintaining the most natural color of dried carrots. Orange juice, which contains carotenoids, natural pigments responsible for red, orange, and yellow colors [64], allows for the formation of dried carrot slices that are most similar to the raw material. Enriching carrot slices with NFC juices can mask visible color changes caused by high drying temperatures. Thermal treatment and drying temperature, depending on the microwave power applied to pineapple slices, caused degradation of pigments, especially carotenoids, and browning resulting from both non-enzymatic Maillard and enzymatic reactions [59].

Considering the sensory qualities of the dried carrots, the panel gave the tested samples a positive and relatively high rating (Table 7). Overall desirability scores were significantly higher for freeze-dried carrots (6.3–7.9) than for MVD (5.5–7.3), mainly due to higher scores for appearance and smell. This may be because of the desirable properties of dried carrot chips, which should feature an appealing appearance, aroma, taste, and texture. High temperatures during drying may promote non-enzymatic browning reactions, such as the Maillard reaction, which may produce volatile compounds, including Strecker aldehydes, primarily responsible for flavor [8]. No significant differences were observed between drying methods in terms of color, crunchiness, and taste. This suggests that the MVD method enables the production of dried products with properties comparable to those of FD. However, compared to FD, the MVD carrot samples studied by Lin et al. [65] scored significantly higher in texture and overall acceptability (5.8–6.6 for FD and 7.8–8.0 for MVD on a 9-point scale), and also in terms of color (7.2 and 7.6) and aroma/taste (6.8 and 7.4).

Instrumentally tested, the hardness and breaking work of MVD-dried samples were significantly higher than those of FD-dried carrots (Table 7). MVD-dried carrots had up to twice the hardness and ten times the breaking work of dried carrot slices. Similarly, in the study by Lin et al. [65], freeze-dried carrot slices showed approximately two times lower hardness values measured by the puncture test than MVD-dried carrots. This may be due to the crispier and more delicate texture of FD-dried carrots.

Osmotic enrichment did not affect appearance, smell, or color. However, most samples, including all those dried by freeze-drying, were rated higher than samples dried without enrichment. The highest taste scores were achieved by samples enriched with carrot juice. Significantly higher crunchiness scores were given to samples enriched with chokeberry, sea buckthorn, and carrot juices. Despite this, instrumental studies did not show a significant effect of enrichment in these juices on the hardness and breaking work of dried carrots.

## 3. Discussion

**Figure 5:**
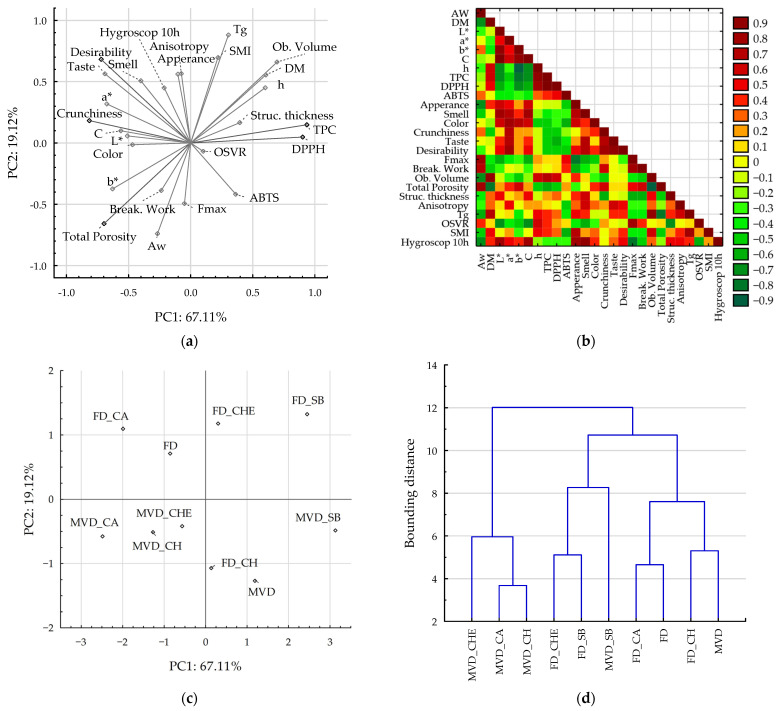
PCA diagram of indicators (**a**) and their heatmap of correlation matrix (**b**) and similarities and differences between dried carrot samples using case projection (**c**) and cluster analysis (**d**).

The research scope, consisting of many key variables related to dried carrot slices, is not easy to interpret and explain physicochemical interdependencies. It is also important to clarify whether indicators of stability, increased chemical component content, and sensory quality of dried carrot slices are interrelated. The technological process parameters and raw material properties discussed in the review article by Santos et al. [66] have attracted intensive research in recent years, primarily focusing on their physicochemical and thermal properties. Compared to fruit, dried vegetables in snack form have not been widely studied, especially in a comprehensive manner, including analysis of thermal properties and microstructure, as well as sensory evaluation. Therefore, principal component analysis (PCA) and correlation matrix are good tools for determining correlations between parameters of dried carrot samples, depending on the initial enrichment in NFC juices and the drying method. Furthermore, case projection and Ward cluster analysis proved useful for identifying similarities and differences between samples (Figure 5). The first two principal components explained 86.33% of the variance of all measured parameters. PC1, explaining 67.11% of the variance, was mainly composed of chemical parameters (TPC polyphenol content and DPPH antioxidant activity), microstructure parameters (total porosity and object volume), most of the characteristics (crunchiness, desirability, taste), and color redness (a*). PC2, on the other hand, consisted mainly of the glass transition temperature (Tg) and water activity (AW).

The PCA variable projection and correlations between the indices resulted in the separation of three clusters in the cluster analysis with a Euclidean distance of nine (Figure 5d). The most different samples were the control samples dried using the MVD method and the chokeberry-enriched MVD_CH. These differences resulted from the highest crunchiness and breaking work of the MVD_CH samples with the lowest MVD indices, which influenced the overall sensory desirability. Compared to the other samples, these samples also showed significant differences in total porosity and OSVR (object surface/volume ratio), as well as hygroscopicity, with values higher for the dried MVD_CH. The first cluster included MVD-dried samples enriched in SB, CHE, and CA juices. Compared to the other two clusters, the mean values of the indices showed the highest water activity, redness, crunchiness, taste, and overall desirability, as well as the indices in the breaking test (Fmax and breaking work), total porosity, and OSVR. This group of samples was characterized by the highest sensory characteristics, of which crunchiness is one of the most important for this type of snack [5,6]. This characteristic was observed in samples with significantly higher water activity and lower glass transition temperature, which contributed to lower stability than other samples.

The high crunchiness scores could be due to the highest total porosity and the greatest number of small pores in the dried carrot slices’ structure. As previously shown (Table 5), regardless of NFC juice, carrot enrichment followed by MVD resulted in increased OSVR, reduced large pores (cavities), and increased total porosity. Similarly, Wang et al. [9] found that the texture of dried peach slices was influenced by the presence of sucrose after osmotic dehydration, which was explained by the strong interaction between sucrose molecules and peach tissue. In the study by Marzec et al. [67], greater crispness correlates with lower water activity. Control samples MVD and FD, and enriched FD_CH and FD_CA in the second cluster, were characterized by the highest color parameters (L*, b*, and C), hence probably the highest distinguishing features in terms of appearance, as well as taste and hygroscopicity. The third group consisted of samples MVD_SB, FD_SB, and FD_CHE, with the lowest water activity and the highest Tg, indicating the highest stability. At the same time, these samples showed the lowest color parameters (except for hue), which were attributed to enrichment with sea buckthorn and cherry juice components. However, these resulted in the lowest sensory parameters, especially cranberry juice and total porosity, but the highest polyphenol content and antioxidant activity (DPPH and ABTS), as well as microstructural indicators such as anisotropy and SMI. Given that all samples received positive sensory parameters, the slightly lower ratings for crispness and other parameters in cluster 3 may be less noticeable when considering sea buckthorn and cherry juices as a valuable source of total phenolic compounds (TPC) and antioxidant properties. Due to the highest polyphenol content and antioxidant activity, low-temperature FD of sea buckthorn juice-enriched samples can be replaced by higher-temperature MVD, primarily because of the significantly shorter process time. PCA and correlation matrices revealed a strong negative correlation between Tg and water activity of the samples, as well as between Tg and total porosity, and a weaker correlation with breaking work. In contrast, enrichment, i.e., polyphenol content and antioxidant activity, had no effect on Tg (correlation ranging from 0.24 to −0.48). At the same time, due to the moderate hygroscopicity of these samples, including lower total porosity, their stability can be maintained under conditions of limited humidity. It was also observed that, despite the highest dry matter content, samples from this cluster had the lowest water activity values, especially those dried using the FD method, whereas in cluster 1, the relationships were reversed. This likely resulted from uneven moisture in the MVD-dried samples or from the interaction between the drying and enrichment methods.

Based on PCA, it was observed that drying method and enriching carrots with NFC juices modify their chemical composition, affecting water activity, glass transition temperature, and short-term hygroscopicity, but have no effect on sorption properties determined by the isotherm method. These changes then translate into textural and sensory characteristics and storage stability of the dried products. Moisture transport and the tissue’s ability to bind water are associated with microstructural properties observed using microtomography and SEM, which can be an indicator of one of the key parameters: the crispness of dried snacks. Dried fruit and vegetable snacks are defined by textural characteristics, especially crispness and crunchiness, which reflect their quality [50,68]. As demonstrated by Peng et al. [69,70], pectin polysaccharides present in the cell walls play a key role in shaping the textural properties of dried carrot chips. Simultaneously, processing can lead to thermal degradation and isomerization of carotenoids [71]. Despite the partial loss of β-carotene during thermal processing, an increase in its bioavailability is observed, associated with its release from the tissue matrix during digestion [72].

## 4. Materials and Methods

### 4.1. Material and Experimental Procedure

The research material consisted of carrots from the “Tadeusz Karaś” horticultural farm, purchased at a large-scale store in Warsaw (Poland) and stored at 2 ± 1 °C in refrigerated conditions. The roots had comparable dimensions with a diameter of about 35 mm and similar color intensity. Half-slices 3 mm thick were used for the tests, obtained with a slicer. All carrot samples were blanched in water at 100 °C for 3 min, then cooled to room temperature. Figure 1 shows a schematic diagram of the experimental procedure for drying carrots.

### 4.2. Technological Methods

#### 4.2.1. Osmotic Enrichment (OE)

Osmotic enrichment of carrots was carried out in NFC (not from concentrate) chokeberry (CH), sea buckthorn (SB), and cherry (CHE) juice produced by Premium Rosa L.L.C. (Złotokłos, Poland), and freshly squeezed carrot juice (CA). The process was carried out in a water bath (JW. Construction, Warsaw, Poland) at 40 °C for 60 min according to the procedure of Ignaczak et al. [5].

#### 4.2.2. Microwave-Vacuum Drying (MVD)

According to the device instructions, carrots were dried using the microwave-vacuum method, preceded by reducing the moisture content of the samples by convection drying at a current airflow of 2.0 ± 0.1 m/s and 60 °C for 30 min in a laboratory convection dryer located at the Institute of Food Sciences at Warsaw University of Life Sciences. Microwave-vacuum drying of carrots was carried out in a PROMIS-TECH LLC dryer (Wrocław, Poland) at a microwave power of 250 W, a pressure of 3.5 kPa, and a maximum vapor temperature at the chamber outlet of 70 °C according to the procedure of Ignaczak et al. [6].

#### 4.2.3. Freeze-Drying (FD)

Freeze drying (FD) of carrot half-slices was carried out after freezing the samples in a shock freezer (Shock Freezer HCM 51.20, Irinox, Treviso, Italy) with airflow at −40 °C for 4 h. The frozen samples were transferred directly to the freeze dryer and dried in an Alpha 1–4 LSC freeze dryer from Christ (Osterode am Harz, Germany) for 24 h at a heating shelf temperature of 30 °C, a pressure inside the chamber of 63 Pa, and a safety pressure of 103 Pa [5].

### 4.3. Analytical Part

#### 4.3.1. Dry Matter Content, Water Activity, and Color Parameters

Dry matter content, water activity, and color parameters were determined according to the procedure described in the previous publication by Ignaczak et al. [5].

#### 4.3.2. Hygroscopicity Determination and Sorption Isotherms

To determine hygroscopicity and sorption isotherm, approximately 1 g of each sample was weighed on an analytical balance (ME54E/M Metler, Warsaw, Poland) to the nearest 0.0001 g. Two replicates were conducted.

Hygroscopic properties (HG) were measured by placing the test samples in a desiccator containing saturated sodium chloride (NaCl) solution at 75% relative humidity and 25  ±  1 °C. The test ran for 72 h. Samples were weighed at 15, 30, and 45 min, and then at 1, 2, 3, 4, 6, 10, 24, 30, 50, and 72 h. Results were expressed as a percentage, calculated by dividing the increase in sample mass during the measurement (m_τ_ − m_o_) by its initial mass (m_o_).

Sorption isotherms (WCeq) were measured using the static desiccator method. Dried samples were placed in 10 desiccators containing saturated salt solutions, maintaining air humidity conditions corresponding to water activities from 0 to 0.93. The desiccators containing the samples were stored for 3 months at a constant 25 °C under atmospheric pressure. In desiccators with high water activity, thymol reduced sample molding. Afterward, the samples were reweighed. The relationships between equilibrium water content and water activity were described using the BET, GAB, Lewicki, Oswin, and Henderson models, which were estimated based on the coefficient of determination (*R*^2^), the percentage root mean square error (*RMSE*), and the residual coefficient of variation (*Ve*). Calculations were performed using the Solver function in Microsoft Excel (MS 365 packages).

#### 4.3.3. Analysis of Texture Properties

The mechanical properties of dried carrots were tested using a TA-HD plus texturometer (Stable Micro Systems, Godalming, UK) according to the procedurę described in Ignaczak et al. [6]. The measurement was performed in at least 10 repetitions. The maximum force obtained during the test (Fmax [N]) and the work mJ [N mm] were determined using the software included with the station, calculated as the product of half the area under the breaking curve and the head speed.

#### 4.3.4. Microstructure Analysis Using Scanning Electron Microscope (SEM)

The structure of dried carrot samples was realized using a Phenom XL scanning electron microscope (Thermo Fisher Scientific, Waltham, MA, USA) according to the procedure described in Ignaczak et al. [6]. At least 5 images of each sample were taken at 500× magnification.

#### 4.3.5. Microstructure and Porosity Analysis Using X-Ray Microtomography

The microstructure of dried carrots was measured using a SkyScan 1272 X-ray micro-CT system (Bruker-microCT, Kontich, Belgium). Dried carrot samples with a height of approximately 20 mm were scanned with a 180° rotation, a 0.4° step, and six frames were averaged at each angular position. The detector-to-source distance was adjusted to achieve a pixel size of 12 μm. Scanning was performed without a filter at 200 μA and 50 kV. A CCD camera with a 9 μm pixel size was used. Image reconstruction was performed using NRecon v1.6.9.8 software (Bruker-microCT, Kontich, Belgium). For quantitative analysis, images were cropped to a region of interest (ROI) of 150 × 30 pixels in CTAn v1.9.3.3 software (Bruker-microCT, Kontich, Belgium). 2D reconstruction was used to generate a 3D model of the sample using v2.3.2.0 CTvox software (Bruker-microCT, Kontich, Belgium). Porosity was calculated from the 3D data using CTAn v.1.10.1.0 software (Bruker, Belgium). Each dried sample was scanned in duplicate. The following microstructural parameters were obtained: percent object volume (POV), closed porosity (CP), total porosity (TP), structure thickness (ST), degree of anisotropy (DA), object surface/volume ratio (OSVR), and structure model index (SMI) [73].

#### 4.3.6. Thermal Properties

The glass transition temperature (Tg) was determined using a differential scanning calorimeter (DSC 3+ STAR, Mettler-Toledo, Greifensee, Switzerland) with liquid nitrogen cooling. Before analysis, the samples were dried in a vacuum oven for 48 h at 30 °C and 10 mbar, then stored over anhydrous P_2_O_5_. A 3–5 mg sample was placed in 50 μL aluminum vessels and subjected to a three-step analysis. Initially, the samples were cooled from 25 to −50 °C, then held at this temperature for 5 min and heated from −50 to 150 °C under a nitrogen atmosphere at a flow rate of 50 mL/min. The cooling rate and N_2_ heating rate were −8 °C/min and 8 °C/min, respectively. DSC curves were analyzed using STAR v.16.0 software [55].

#### 4.3.7. Fourier Transform Infrared (FTIR) Spectroscopy

The chemical composition and molecular structure of dried half-slices of carrots were determined using Fourier transform infrared spectroscopy [19]. The analysis was performed in the wavelength range of 650–4000 cm^−1^, with a resolution of 4 cm^−1^, using 32 scans of the spectrum with a Cary 630 spectrometer with MicroLab FTIR software version 5.7 (Agilent Technologies Inc., Santa Clara, CA, USA). The attenuated total reflection (ATR) sensor used a single reflection interface, which was a diamond crystal. The previously ground samples in powder form were pressed against the crystal using a pressure clamp. A background spectrum was collected before each scan of the sample. The analysis was performed in triplicate.

### 4.4. Chemical Assays

#### 4.4.1. Extraction Procedure

Extracts for chemical determinations were prepared according to the methodology described by Ignaczak et al. [6] using 80% (*v*/*v*) aqueous ethanol solution.

#### 4.4.2. Total Phenolic Content (TPC) Determination

The total phenolic content (TPC) was determined spectrophotometrically using the Multiskan Sky plate reader (Thermo Electron Co., Waltham, MA, USA) and the Folin–Ciocalteu reagent according to the procedure described in Ignaczak et al. [6]. The total phenolic content (TPC) was calculated from a calibration curve prepared with gallic acid, and the results were expressed as mg of gallic acid equivalents (GAE) per 100 g of dry matter. The determination was performed in triplicate for each extract.

#### 4.4.3. Antioxidant Activity Determination

The antioxidant activity of dried carrot samples was determined spectrophotometrically using the Multiskan Sky plate reader (Thermo Electron Co., Waltham, MA, USA) and the DPPH• and ABTS•+ radical solutions according to the procedure described in Ignaczak et al. [6]. Results are expressed in mg Trolox (TE) per g dry matter (d.m.). The analysis was performed in triplicate for each extract.

### 4.5. Sensory Evaluation

Sensory evaluation of dried carrot samples prepared 3–4 days earlier and marked with appropriate codes was performed by 10 people aged 21–58, including students and employees of the University of Life Sciences in Warsaw, according to the methodology described by Ignaczak et al. [6]. A 9-point hedonic scale was used (like extremely—9, like very much—8, like moderately—7, like slightly—6, neither like nor dislike—5, dislike slightly—4, dislike moderately—3, dislike very much—2, and dislike extremely—1). The study did not require formal ethical approval. Panel members were informed about the testing methodology, samples, and how the data would be used for research purposes. All provided verbal consent to participate in the sensory evaluation voluntarily and could withdraw at any time. Panelists did not experience any danger or discomfort while testing the samples. The rights and privacy of participants were safeguarded by Regulation (EU) 679/2016. The assessors were informed of the distinguishing features and threshold values, including appearance, smell, color, crunchiness, flavor, and overall desirability. The carrot snacks were labeled with codes and served on plastic trays. Fresh, non-carbonated water was supplied to rinse the mouth. The assessors completed the survey anonymously.

### 4.6. Statistical Analysis

The results were statistically analyzed using Statistica 13.3 PL (TIBCO Software Inc, StatSoft, Cracow, Poland), Microsoft Excel (MS 365 packages), and MS ver. 2019. Analysis of variance ANOVA was used to assess the influence of the studied factors, drying method, and enriching, at a significance level of 0.05. Repeated measurements (ANOVA) designs were used to calculate significant differences between hygroscopicity and sorption isotherms. The Tukey HSD test was used to identify homogeneous groups. Pearson’s correlation and principal component analysis (PCA) were performed to examine the relationships among indicators. Warda’s clustering and heatmap with a correlation matrix were employed to investigate the similarity between data groups and samples. Experiments included at least 2 replicates. Data are presented as means ± standard deviation.

## 5. Conclusions

The specific structure of the root, related to its transport function, favors both the enrichment of the raw material with additional ingredients and the drying processes, while also enabling the shaping of the sensory properties and stability of the dried product. Enriching carrots in NFC juices, which are carriers of biologically active compounds, allows for obtaining dried products with an increased content of total polyphenols and shaping the sensory properties of dried carrots, especially crunchiness, one of the most important characteristics of snacks, as well as appearance. At the same time, the selection of microwave-vacuum drying conditions, including pre-treatment, influenced the water content and microstructure, as well as the thermal properties of the dried carrots.

Microwave-vacuum drying (MVD), like freeze-drying (FD), affected product stability. Pre-enrichment of carrots with juices, especially chokeberry and cherry, increased stability and natural chemical components content and sensory properties, as confirmed by lower water activity and an approximately 10-fold increase in polyphenol content relative to the raw material. Although the antioxidant activity of dried samples enriched with chokeberry and cherry juices was lower than that of the raw material, enrichment with these juices significantly increased it relative to the control samples and to those enriched with other juices. The higher glass transition temperature of the FD samples (average 62.0 °C) indicated their greater stability than MVD (56 °C). The highest Tg was obtained for samples enriched with chokeberry juice (FD: 69.6 °C; MVD: 58.7 °C), and the lowest for sea buckthorn (52.2–53.7 °C).

Drying and enrichment with NFC juices influenced the sorption properties of dried carrots. While hygroscopicity was tested in a short period (three days) under high humidity conditions, which depended on the drying method, equilibrium moisture content after three months depended on juice enrichment. Due to compositional changes during storage, sorption isotherms did not correlate with hygroscopicity. Samples enriched with chokeberry juice exhibited high hygroscopicity, similar to carrots dried by the FD method, while MVD samples enriched with SB juice were the most stable. Simultaneously, their sorption isotherms achieved the highest equilibrium moisture values. Higher water sorption capacity was associated with smaller microstructural changes and greater porosity in freeze-dried samples (numerous small, open pores), whereas large pores dominated in MVD samples. Regardless of the juice type, MVD enrichment and drying resulted in a more uniform structure, a greater number of small pores, fewer cavities, and increased overall porosity. The tested samples exhibited similar spectral shapes in FTIR analysis, revealing similar chemical composition and functional group distribution, with simultaneous differences in peak intensities.

Based on the above studies and previous ones [5,6], analyzing the prospects for carrot processing into dried snacks, it can be predicted that the use of simple carrot processing methods, including enrichment in NFC juices and short treatment at elevated temperatures in microwave-vacuum drying, will replace the freeze-drying method, resulting in a high-quality product in terms of natural components, including carotenoids, polyphenols, vitamins, sugars, fiber, and others, which positively impact texture and overall desirability, as well as stability in terms of water content and other aspects. This innovative solution will enable the production of dried carrots and other vegetable snacks that satisfy consumer preferences.

## Figures and Tables

**Table 1:** Dry matter content (DM) and water activity (AW), total polyphenol content (TPC), and antioxidant activity (DPPH, ABTS) of dried carrot, depending on drying method and osmotic enrichment in NFC juices.

Sample Codes	Dry Matter Content, DM [%]	Water Activity, AW [-]	TPC [mg GAE/100 g d.m.]	DPPH [mg Trolox/g d.m.]/ABTS [mg Trolox/g d.m.]
Fresh Carrot	12.40 ± 0.14	0.986 ± 0.002	235.8 ± 62.6 ^aA^	5.2 ± 1.1/4.6 ± 0.5
MVD	89.83 ± 1.58 ^aA^	0.459 ± 0.001 ^bA^	1194.3 ± 102.3 ^aB^	4.6 ± 0.6 ^aB^/3.5 ± 0.8 ^bB^
MVD_CH	96.91 ± 0.24 ^aB^	0.242 ± 0.005 ^bA^	2350.4 ± 198.6 ^aD^	6.4 ± 1.0 ^aD^/4.9 ± 0.1 ^bB^
MVD_SB	91.01 ± 1.66 ^aA^	0.477 ± 0.005 ^bA^	779.4 ± 66.6 ^aB^	3.7 ± 0.2 ^aA^/2.8 ± 0.3 ^bB^
MVD_CHE	89.79 ± 0.62 ^aA^	0.392 ± 0.003 ^bA^	1345.3 ± 39.5 ^aC^	4.8 ± 0.4 ^aC^/4.5 ± 0.4 ^bC^
MVD_CA	89.64 ± 0.84 ^aA^	0.477 ± 0.004 ^bA^	345.4 ± 26.6 ^aA^	0.9 ± 0.7 ^aA^/1.9 ± 0.2 ^bA^
FD	93.70 ± 1.08 ^bA^	0.128 ± 0.003 ^aA^	752.9 ± 41.2 ^aB^	2.4 ± 0.4 ^aB^/2.9 ± 0.4 ^aB^
FD_CH	95.60 ± 0.40 ^bB^	0.092 ± 0.003 ^aA^	2443.2 ± 132.9 ^aD^	7.5 ± 0.2 ^aD^/0.9 ± 0.1 ^aB^
FD_SB	92.28 ± 0.31 ^bA^	0.159 ± 0.001 ^aA^	922.4 ± 40.4 ^aB^	4.1 ± 0.2 ^aB^/3.3 ± 0.8 ^aB^
FD_CHE	93.22 ± 0.31 ^bA^	0.114 ± 0.002 ^aA^	1453.2 ± 39.6 ^aC^	5.3 ± 0.6 ^aC^/4.5 ± 0.5 ^aC^
FD_CA	93.85 ± 0.03 ^bA^	0.096 ± 0.001 ^aA^	235.5 ± 40.0 ^aA^	0.7 ± 0.1 ^aA^/0.6 ± 0.2 ^aA^

Explanations: homogeneous groups at *p* = 0.05; ^a^, ^b^—effect of drying method: MVD—microwave-vacuum drying; FD—freeze-drying; ^A^, ^B^, ^C^, ^D^—effect of osmotic enrichment in NFC juices: CH—chokeberry juice; SB—sea buckthorn; CHE—cherry juice; CA—carrot juice.

**Table 2:** Statistical analysis of dried carrot hygroscopicity and sorption isotherms, depending on osmotic enriching (pretreatment), drying method, and their cooperation.

Hygroscopicity			Sorption Isotherms		
Factors/*p*-Value		Medium Value	Factors/*p*-Value		Medium Value
MVD control ^a^ CH ^a^ SB ^a^ CHE ^a^ CA ^a^ FD control ^a^	Pretreatment/0.2015	7.1624 10.2508 7.8330 8. 119 8.7986 10.6442	MVD control ^a^ CH ^d^ SB ^f^ CHE ^b^ CA ^c^ FD control ^d^	Pretreatment/* 0.0000	14.4830 18.9551 18.6074 16.1130 17.6473 18.1058
MVD ^a^ FD ^b^	Drying method/* 0.0021	7.5403 9.9787	MVD ^b^ FD ^a^	Drying method/* 0.0000	17.6512 17.3957
15 ^a^ 30 ^a^ 45 ^ab^ 60 ^ab^ 120 ^bc^ 180 ^cd^ 240 ^de^ 360 ^e^ 600 ^f^ 1440 ^g^ 1800 ^g^ 3000 ^h^ 4320 ^h^	Time/* 0.0000	1.35906 1.36176 1.59880 1.79313 3.01804 3.85984 4.74994 6.10045 9.45298 17.62265 17.91786 22.22446	0.000 ^b^ 0.113 ^a^ 0.225 ^c^ 0.329 ^d^ 0.438 ^e^ 0.529 ^f^ 0.648 ^g^ 0.753 ^i^ 0.810 ^j^ 0.930 ^k^	Water activity/* 0.0000	0.1407 0.2052 1.1409 2.9711 5.7259 8.1854 15.2971 20.3506 29.0330 92.1845
many cases	Pretreatment × Time * 0.0359	many cases	many cases	Pretreatment × Water activity * 0.0000	many cases
many cases	Drying method × Time * 0.0359	many cases	many cases	Drying method × Water activity * 0.0000	many cases

Explanations: *—means statistically significant differences at *p* = 0.05; ^a–k^—homogeneous groups regarding the influence of factors, respectively pretreatment, drying method and time: MVD—microwave-vacuum drying; FD—freeze-drying, CH—chokeberry juice; SB—sea buckthorn; CHE—cherry juice; CA—carrot juice.

**Table 3:** Parameters of models used to describe sorption isotherms of dried carrot and fit indicators.

Models/Parameters		MVD	MVD_CH	MVD_SB	MVD_CHE	MVD_CA	FD	FD_CH	FD_SB	FD_CHE	FD_CA
	*Xm*	6.059	6.976	7.164	6.929	8.124	7.280	6.820	7.278	6.103	6.454
BET	*C*	0.820	1.394	1.335	0.902	0.715	0.984	2.233	0.985	0.978	0.918
	*R* ^2^	0.999	0.998	0.999	0.998	0.999	0.998	0.999	0.999	0.999	0.995
	*RMSE*	0.739	1.282	0.962	1.071	1.094	1.108	1.033	1.013	0.861	1.751
	*Ve*	6.096	8.272	6.057	7.506	6.804	7.315	6.370	6.636	6.785	13.014
	*Xm*	7.265	6.970	6.807	6.095	6.514	7.376	7.242	6.719	7.125	5.024
GAB	*C*	0.566	1.398	1.559	1.276	1.290	0.953	1.826	1.221	0.694	2.016
	*K*	0.990	1.000	1.003	1.007	1.012	0.999	0.996	1.005	0.991	1.014
	*R* ^2^	0.999	0.998	0.999	0.999	0.999	0.998	0.999	0.999	0.999	0.996
	*RMSE*	0.665	1.282	0.938	0.992	0.853	1.108	0.987	0.975	0.790	1.520
	*Ve*	5.486	8.272	5.906	6.947	5.308	7.310	6.086	6.387	6.224	11.301
	*a*	4.809	6.487	6.337	5.043	4.984	5.972	7.699	5.671	5.330	4.371
Lewicki	*b*	1.066	1.021	1.039	1.101	1.157	1.059	0.956	1.078	1.036	1.129
	*c*	3.922	1.808	1.606	1.911	1.560	2.596	1.487	2.055	3.460	1.166
	*R* ^2^	0.999	0.998	0.999	0.999	0.999	0.999	0.999	0.999	0.999	0.996
	*RMSE*	0.720	1.230	0.891	0.954	0.853	1.033	0.915	0.910	0.785	1.580
	*Ve*	5.943	7.939	5.612	6.684	5.307	6.818	5.641	5.961	6.187	11.743
Oswin	*a*	5.601	7.902	7.940	6.556	6.893	7.237	9.030	7.177	6.111	6.056
	*b*	1.024	0.959	0.967	1.019	1.054	1.002	0.905	1.005	0.998	1.023
	*R* ^2^	0.999	0.998	0.999	0.998	0.999	0.998	0.999	0.999	0.999	0.995
	*RMSE*	0.773	1.300	1.013	1.050	0.996	1.109	1.019	1.009	0.862	1.722
	*Ve*	5.970	7.847	5.967	6.882	5.797	6.845	5.875	6.184	6.355	11.975
Henderson	*a*	0.389	0.311	0.315	0.365	0.374	0.340	0.269	0.345	0.364	0.384
	*b*	0.440	0.472	0.467	0.440	0.422	0.451	0.506	0.448	0.454	0.436
	*R* ^2^	0.998	0.992	0.992	0.993	0.994	0.995	0.992	0.994	0.997	0.987
	*RMSE*	1.128	2.415	2.434	2.193	2.408	2.002	2.477	2.219	1.325	2.786
	*Ve*	8.707	14.578	14.341	14.372	14.013	12.359	14.284	13.605	9.770	1.931

Explanations: *Xm*—monolayer water content [g/100 g d.m.] as a parameter of the BET model, *C*, *K*, *a*, *b*, *c*—constants of model equations, *R*^2^—coefficient of determination *RMSE*—root mean square error, *Ve*—coefficient of residual variation.

**Table 4:** Photos of overall appearance (section A) and SEM imaging of the internal structure at magnification 500× (section B), and µCT reconstruction of the internal structure 3D (section C) of microwave-vacuum and freeze-dried carrot, depending on drying method and osmotic enrichment in NFC juices.

Sample	A	B	C
MVD	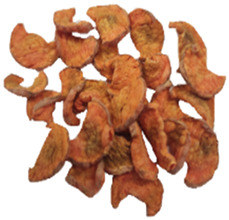	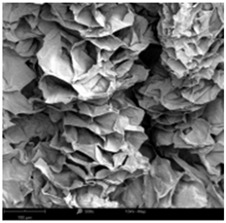	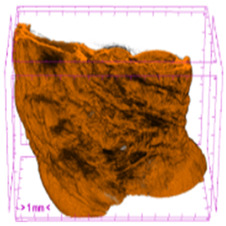
MVD_CH	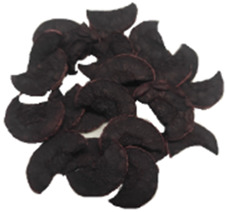	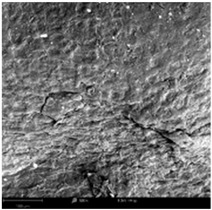	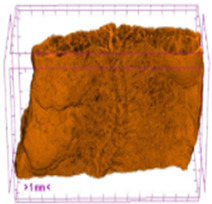
MVD_SB	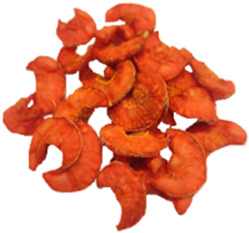	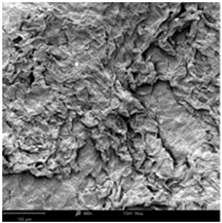	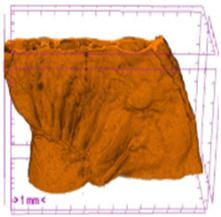
MVD_CHE	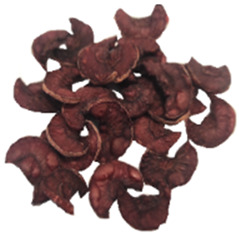	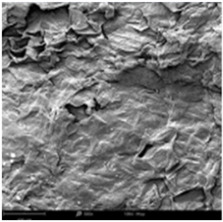	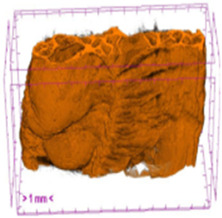
MVD_CA	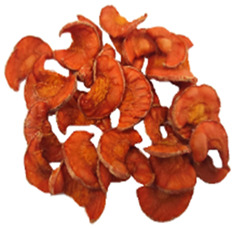	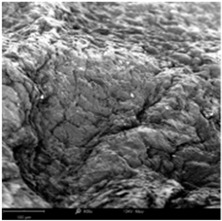	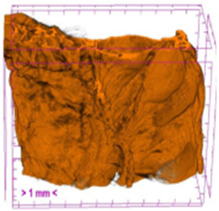
FD	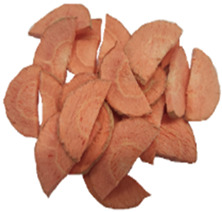	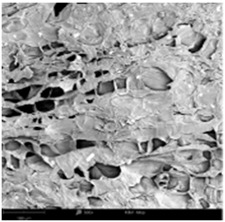	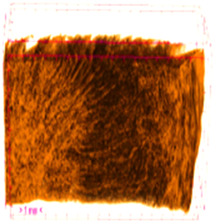
FD_CH	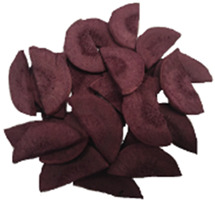	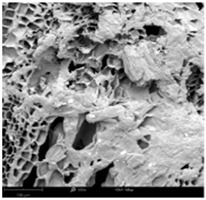	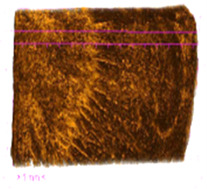
FD_SB	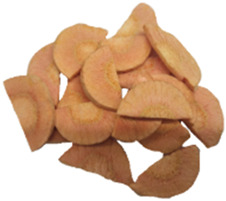	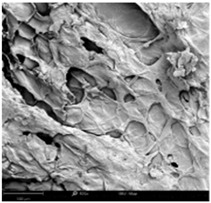	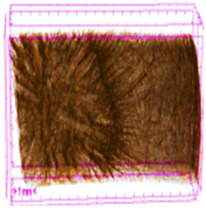
FD_CHE	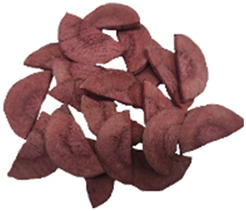	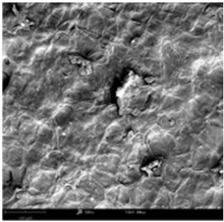	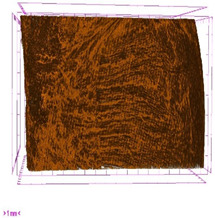
FD_CA	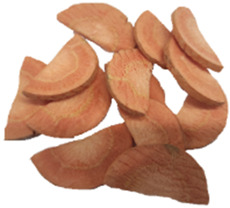	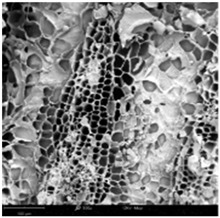	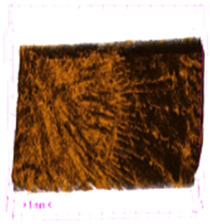

**Table 5:** 3D microstructural parameters of microwave-vacuum and freeze-dried carrot, depending on drying method and osmotic enrichment in NFC juices.

Sample Codes	Percent Object Volume [%] POV	Closed Porosity [%] CP	Total Porosity [%] TP	Structure Thickness [mm] ST	Degree of Anisotropy [−] DA	Object Surface/Volume Ratio [mm^−1^] OSVR	Structure Model Index SMI
MVD	18.07 ± 1.31 ^aAB^	0.17 ± 0.01 ^aB^	81.51 ± 0.71 ^bAB^	0.041 ± 0.004 ^aA^	1.79 ± 0.04 ^aA^	91.97 ± 0.77 ^bA^	0.65 ± 0.01 ^aA^
MVD_SB	11.48 ± 1.37 ^aA^	0.03 ± 0.00 ^aA^	88.52 ± 1.37 ^bB^	0.032 ± 0.002 ^aA^	2.14 ± 0.99 ^aA^	133.60 ± 1.66 ^bC^	0.58 ± 0.01 ^aC^
MVD_CHE	11.33 ± 0.36 ^aA^	0.03 ± 0.02 ^aA^	88.67 ± 0.36 ^bAB^	0.031 ± 0.004 ^aA^	1.81 ± 0.32 ^aA^	106.89 ± 1.05 ^bBC^	0.60 ± 0.01 ^aAB^
MVD_CA	7.44 ± 2.49 ^aA^	0.06 ± 0.01 ^aA^	92.56 ± 2.49 ^bB^	0.031 ± 0.003 ^aA^	2.91 ± 0.39 ^aA^	133.06 ± 19.73 ^bC^	0.74 ± 0.12 ^aBC^
MVD_CH	29.83 ± 0.81 ^aB^	0.04 ± 0.00 ^aA^	70.95 ± 0.48 ^bA^	0.034 ± 0.001 ^aA^	1.84 ± 0.02 ^aA^	134.37 ± 20.95 ^bAB^	0.65 ± 0.19 ^aA^
FD	23.31 ± 1.86 ^bAB^	0.14 ± 0.01 ^aB^	76.69 ± 1.86 ^aAB^	0.040 ± 0.003 ^bA^	3.26 ± 0.48 ^aA^	82.40 ± 1.52 ^aA^	2.25 ± 0.02 ^bA^
FD_SB	13.35 ± 0.55 ^bA^	0.01 ± 0.01 ^aA^	85.72 ± 1.86 ^aB^	0.038 ± 0.001 ^bA^	1.91 ± 0.01 ^aA^	121.36 ± 1.72 ^aC^	0.77 ± 0.13 ^bC^
FD_CHE	25.44 ± 0.10 ^bA^	0.06 ± 0.00 ^aA^	74.56 ± 0.10 ^aAB^	0.037 ± 0.001 ^bA^	2.36 ± 0.28 ^aA^	89.26 ± 1.74 ^aBC^	1.14 ± 0.05 ^bAB^
FD_CA	23.37 ± 2.91 ^bA^	0.01 ± 0.01 ^aA^	76.63 ± 2.91 ^aB^	0.035 ± 0.000 ^bA^	2.00 ± 0.26 ^aA^	97.01 ± 0.62 ^aC^	1.63 ± 0.51 ^bBC^
FD_CH	33.68 ± 1.96 ^bB^	0.04 ± 0.01 ^aA^	66.32 ± 1.96 ^aA^	0.038 ± 0,006 ^bA^	3.36 ± 0.49 ^aA^	107.02 ± 0.64 ^aAB^	0.65 ± 0.01 ^bA^

Explanations: homogeneous groups at *p* ≤ 0.05: ^a^, ^b^—effect of drying method: MVD—microwave-vacuum drying; FD—freeze-drying; ^A^, ^B^, ^C^—effect of osmotic enrichment in NFC juices: CH—chokeberry juice; SB—sea buckthorn; CHE—cherry juice; CA—carrot juice.

**Table 6:** Color parameters of dried carrot, depending on drying method and osmotic enrichment in NFC juices.

Sample Code	Parameter L*	Parameter a*	Parameter b*	Saturation C	Hue Color h	ΔE
Fresh Carrot	35.7 ± 0.1 ^aA^	13.8 ± 0.1 ^aAB^	23.8 ± 0.1 ^aC^	38.3 ± 0.1 ^aC^	30.0 ± 0.1 ^aA^	-
MVD	50.2 ± 3.3 ^aB^	14.8 ± 2.4 ^aBC^	38.4 ± 2.5 ^aD^	52.3 ± 1.3 ^aD^	21.0 ± 2.0 ^aA^	20.7 ± 3.9 ^aB^
MVD_SB	44.3 ± 3.0 ^aB^	25.6 ± 2.1 ^aC^	38.9 ± 3.7 ^aA^	51.2 ± 0.8 ^aD^	33.5 ± 3.3 ^aA^	21.2 ± 3.8 ^aB^
MVD_CHE	52.4 ± 4.2 ^aA^	22.9 ± 3.1 ^aBC^	48.8 ± 3.3 ^aB^	30.0 ± 1.6 ^aB^	65.1 ± 2.3 ^aB^	26.7 ± 5.0 ^aA^
MVD_CA	54.0 ± 2.8 ^aB^	14.2 ± 1.9 ^aC^	37.4 ± 1.9 ^aD^	47.3 ± 1.3 ^aA^	34.3 ± 2.0 ^aA^	17.8 ± 2.5 ^aAB^
MVD_CH	20.5 ± 1.0 ^aA^	4.61 ± 0.7 ^aA^	1.4 ± 0.5 ^aA^	21.0 ± 1.5 ^aD^	73.6 ± 1.0 ^aC^	28.6 ± 1.1 ^aAB^
FD	71.2 ± 1.81 ^bB^	23.4 ± 2.5 ^bBC^	36.5 ± 1.6 ^aD^	75.0 ± 3.6 ^aD^	32.5 ± 1.8 ^aA^	22.4 ± 0.8 ^bB^
FD_SB	68.1 ± 0.9 ^bB^	20.4 ± 1.9 ^bC^	42.6 ± 3.7 ^aA^	71.1 ± 2.8 ^aD^	25.7 ± 3.3 ^aA^	38.2 ± 1.5 ^bB^
FD_CHE	25.9 ± 2.5 ^bA^	14.9 ± 1.4 ^bBC^	6.9 ± 0.7 ^aB^	45.5 ± 2.4 ^aB^	69.8 ± 2.7 ^aB^	19.7 ± 1.6 ^bA^
FD_CA	40.8 ± 2.5 ^bB^	23.6 ± 2.8 ^bC^	34.7 ± 2.2 ^aD^	72.6 ± 1.8 ^aA^	28.5 ± 4.7 ^aA^	15.9 ± 1.2 ^bAB^
FD_CH	29.7 ± 1.7 ^bA^	19.9 ± 0.7 ^bA^	3.2 ± 0.3 ^aA^	35.8 ± 1.1 ^aD^	80.9 ± 4.1 ^aC^	17.6 ± 8.8 ^bAB^

Explanations: homogeneous groups at *p* = 0.05: ^a^, ^b^—effect of drying method: MVD—microwave-vacuum drying; FD—freeze-drying; ^A^, ^B^, ^C^, ^D^—effect of osmotic enrichment in NFC juices: CH—chokeberry juice; SB—sea buckthorn; CHE—cherry juice; CA—carrot juice, ΔE—absolute color difference.

**Table 7:** Sensory evaluation results using a 9-point hedonic scale and mechanical properties of dried carrots depending on the drying method and osmotic enrichment in NFC juices.

Sample Code	Appearance	Smell	Color	Crunchiness	Taste	Overall Desirability	Hardness; Fmax [N]	Breaking Work [mJ]
MVD	5.6 ± 1.2 ^aA^	6.3 ± 2.2 ^aA^	7.0 ± 1.6 ^aA^	5.2 ± 1.0 ^aA^	6.4 ± 1.6 ^aAB^	5.8 ± 1.5 ^aAB^	7.9 ± 1.4 ^bA^	3.1 ± 0.8 ^bA^
MVD_SB	6.1 ± 1.5 ^aA^	6.1 ± 1.9 ^aA^	7.9 ± 1.0 ^aA^	8.4 ± 0.8 ^aB^	6.7 ± 0.9 ^aA^	6.8 ± 0.9 ^aA^	6.9 ± 0.8 ^bA^	8.6 ± 0.3 ^bA^
MVD_CHE	5.4 ± 1.3 ^aA^	6.2 ± 2.2 ^aA^	6.5 ± 1.5 ^aA^	8.2 ± 0.8 ^aB^	6.8 ± 1.8 ^aAB^	6.8 ± 1.4 ^aAB^	11.6 ± 2.3 ^bA^	11.1 ± 0.3 ^bA^
MVD_CA	6.1 ± 1.6 ^aA^	6.5 ± 1.9 ^aA^	7.6 ± 1.2 ^aA^	8.0 ± 0.8 ^aB^	7.8 ± 1.8 ^aB^	7.3 ± 1.5 ^aB^	9.4 ± 1.5 ^bA^	3.0 ± 0.5 ^bA^
MVD_CHE	5.4 ± 1.3 ^aA^	6.2 ± 2.2 ^aA^	6.5 ± 1.5 ^aA^	8.2 ± 0.8 ^aB^	6.8 ± 1.8 ^aAB^	6.8 ± 1.4 ^aAB^	11.6 ± 2.3 ^bA^	11.1 ± 0.3 ^bA^
MVD_CH	5.5 ± 1.6 ^aA^	5.6 ± 1.6 ^aA^	6.2 ± 1.8 ^aA^	4.9 ± 1.0 ^aA^	5.7 ± 1.8 ^aA^	5.5 ± 1.4 ^aA^	8.9 ± 1.6 ^bA^	3.1 ± 0.1 ^bA^
FD	6.7 ± 1.3 ^bA^	7.4 ± 1.3 ^bA^	7.0 ± 1.6 ^aA^	7.0 ± 1.4 ^aA^	7.9 ± 1.0 ^aAB^	7.4 ± 0.8 ^bAB^	4.8 ± 1.1 ^aA^	2.0 ± 1.2 ^aA^
FD_SB	8.1 ± 0.9 ^bA^	6.7 ± 1.3 ^bA^	8.0 ± 1.2 ^aA^	6.1 ± 1.1 ^aB^	5.6 ± 1.7 ^aA^	6.3 ± 1.2 ^bA^	4.1 ± 0.9 ^aA^	1.0 ± 1.3 ^aA^
FD_CHE	7.4 ± 0.8 ^bA^	6.8 ± 1.3 ^bA^	7.0 ± 1.3 ^aA^	7.0 ± 1.1 ^aB^	7.5 ± 1.1 ^aAB^	7.5 ± 1.1 ^bAB^	5.2 ± 0.6 ^aA^	1.2 ± 2.7 ^aA^
FD_CA	8.0 ± 0.8 ^bA^	6.7 ± 1.7 ^bA^	7.5 ± 1.2 ^aA^	7.4 ± 1.3 ^aB^	7.8 ± 1.1 ^aB^	7.9 ± 1.0 ^bB^	5.1 ± 1.2 ^aA^	1.3 ± 0.4 ^aA^
FD_CH	7.7 ± 1.3 ^bA^	6.6 ± 1.4 ^bA^	7.5 ± 1.4 ^aA^	6.3 ± 1.1 ^aA^	6.6 ± 1.1 ^aA^	6.8 ± 0.8 ^bA^	4.8 ± 0.9 ^aA^	0.7 ± 1.5 ^aA^

Explanations: homogeneous groups at *p* = 0.05: ^a^, ^b^—effect of drying method: MVD—microwave-vacuum drying; FD—freeze-drying; ^A^, ^B^—effect of osmotic enrichment in NFC juices: CH—chokeberry juice; SB—sea buckthorn; CHE—cherry juice; CA—carrot juice.

## Data Availability

The original contributions presented in this study are included in the article. Further inquiries can be directed to the corresponding authors.
